# Bacterial resistance to antibiotic alternatives: a wolf in sheep’s clothing?^1^

**DOI:** 10.1093/af/vfy003

**Published:** 2018-04-28

**Authors:** Benjamin P Willing, Deanna M Pepin, Camila S Marcolla, Andrew J Forgie, Natalie E Diether, Benjamin C T Bourrie

**Affiliations:** Department of Agricultural, Food and Nutritional Science, University of Alberta, Edmonton, Alberta, Canada

**Keywords:** antibiotic alternatives, resistance mechanisms, responsible use

**Implications**

- Substantial pressure to reduce antibiotic use has necessitated the development of antibiotic alternatives. However, relatively little consideration has been given to the development of resistance to these alternatives.
- Whether we come up with antibiotic alternatives that are bacteriocidal or inhibitory, bacteria will continue to adapt and evolve.
- Some antibiotic alternatives support the development of antibiotic resistance necessitating caution.
- There are opportunities to optimize antibiotic alternative effectiveness as well as to minimize the development of resistance mechanisms.

## Introduction

**Figure 1.**
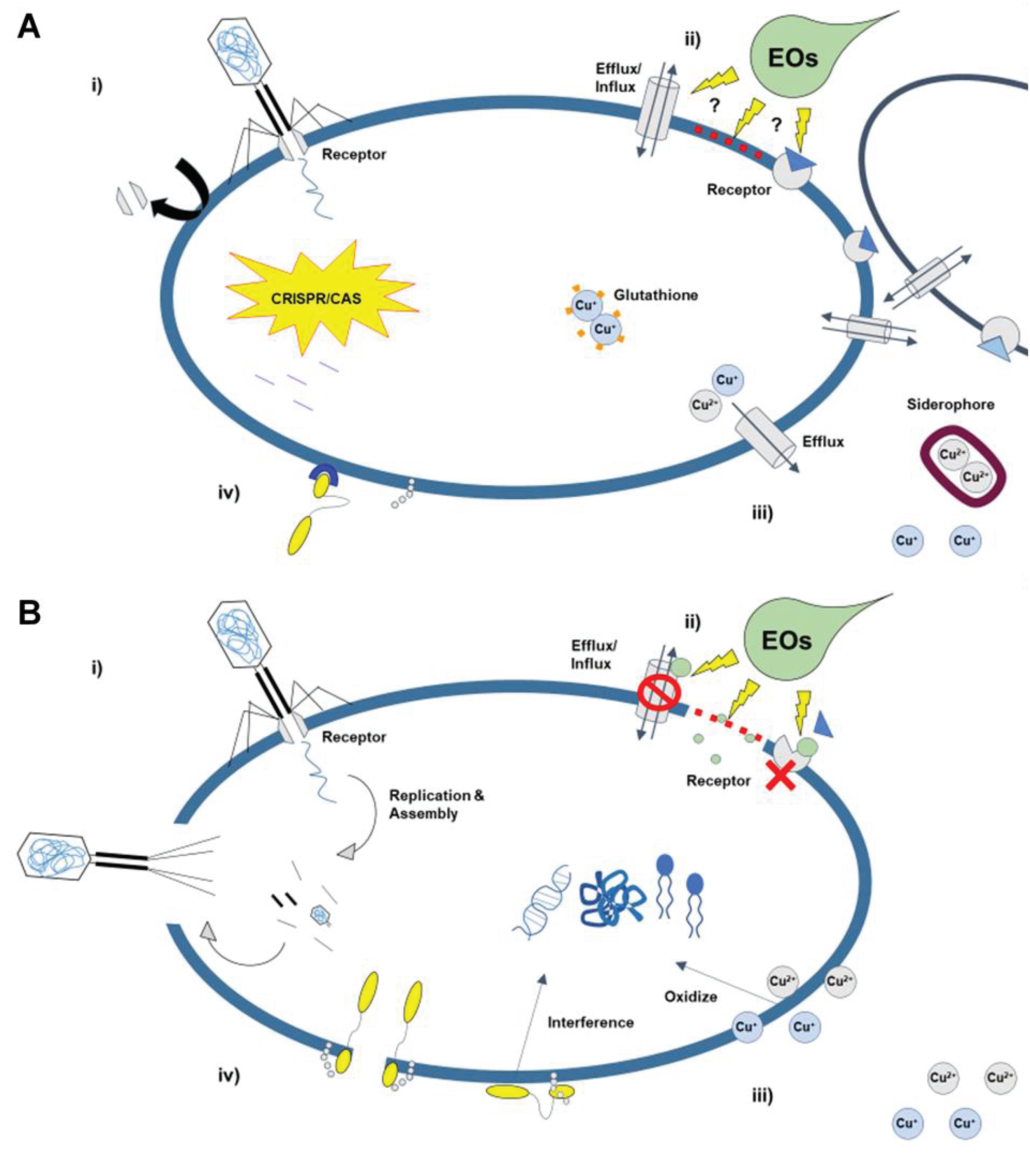
(A) Phage interact with specific receptors to inject DNA into the bacterial cell, causing viral proliferation and cell lysi (i). Essential oils (**EO**s) disrupt efflux/influx, membrane receptors and stability (ii). Copper disrupts bacterial lipids, proteins, and DNA through oxidization (iii). Bacteriocins cause cell wall lysis, disrupt the plasma membrane structure (pore formation), and interfere with DNA function (iv). (B) Bacterial resistance to phage is conferred through either blockage/removal of the receptor or cutting of phage DNA in the cell by CRISPR/CAS (i). Bacteria form aggregates to minimize cell surface exposure to EOs, thus preventing membrane associated disruptions (ii). Glutathione chelates Cu^+^, ATPase efflux system exports Cu^+^/Cu^2+^, and siderophores sequester Cu^2+^ to prevent it entering the cell (iii). Modifications of the cell wall and membrane affect fluidity and charge, impairing bacteriocin binding (iv).

With the growing concern of antibiotic resistance (Aminov and Mackie, 2007; Zaman et al., 2017), there has been a strong push to reduce the use of antibiotics in animal production systems (Van Boeckel et al., 2015; Ventola, 2015). Many antibiotic alternatives have been developed, with varying degrees of success in improving health outcomes and growth performance (Gresse et al., 2017). These alternatives use very different approaches to regulate both commensal and pathogenic bacterial populations. Antibiotic alternatives such as phage and bacteriocins have very clear mechanisms of antimicrobial activity (Figure 1), whereas others, such as essential oils/phytosterols, have less defined modes of action. Irrespective of mode of action, there has been insufficient attention given to the ability of bacteria to develop resistance to these antibiotic alternatives. Considering the development of resistance will be essential in finding long-term solutions. In this review, we present what is known about the ability of bacteria to become resistant to these antibiotic alternatives, and more importantly, identify where they contribute to antibiotic resistance. Prudence is required, as avoiding further contribution to antibiotic resistance is necessary. This review is not exhaustive but is intended to give a good representation from different classes of antibiotic alternatives. In particular, we focus on phage, essential oils, direct-fed microbials and bacteriocins, metals and minerals, and organic acids. Some consideration is given to their application, effectiveness, and modes of action.

### Bacteriophages

Bacteriophages are viruses that can infect and kill bacteria. In the environment, there is a constant arms race between bacteria and phages: as bacteria develop resistance mechanisms, new phages emerge. Bacteriophages are highly specific, which makes them intriguing antibiotic alternatives as they are less likely to affect commensal bacteria. Although, bacteriophages have not yet been widely adopted in animal production systems, they have been shown to be effective in controlling pathogenic bacteria in feed animals. Research has shown potential for phages to control colonization of *Campylobacter jejuni* (Carrillo et al., 2005), and *Salmonella* (Borie et al., 2008; Bardina et al., 2012), while decreasing mortality in chickens during *Escherichia coli* infection (Huff et al., 2002; Huff et al., 2006). Phages have also been effective in reducing *Salmonella* shedding in pigs (Saez et al., 2011) and reducing shedding of *E. coli* O157:H7 in sheep (Bach et al., 2009; Raya et al., 2011). However, efforts targeting *E. coli* O157 in cattle have proven to be less successful (Rozema et al., 2009; Rivas et al., 2010; Stanford et al., 2010).

While phages present potential for the control of pathogenic bacteria, there are significant considerations still required regarding their implementation in animal production. As with antibiotics, bacteria are capable of developing resistance to phage infection utilizing systems such as the Clustered Regularly Interspaced Short Palindromic Repeat (**CRISPR**) system as a pseudoimmune system, or the abortive infection system to kill infected cells before the phage can spread (Labrie et al., 2010). Additionally, bacteria can alter their cell surface to remove or block the receptor to which the phage binds (Labrie et al., 2010), which can impact virulence or colonization factors (Cryz et al., 1984). For example, the occurrence of phage-resistant *C. jejuni* has been noted; however, all isolates exhibited decreased ability to colonize the cecum of broiler chickens (Carrillo et al., 2005). As resistance to multiple phages can be difficult for bacteria to develop, the use of phage cocktails which target different receptors is recommended. This has been shown to result in superior reduction of bacterial cells with fewer incidence of resistant strains and is commonly used in studies examining phage treatment (Huff et al., 2002; Carrillo et al., 2005; Rivas et al., 2010).

Another factor to consider in the use of phages is the characteristics of the phages themselves. Phages can be both lytic and lysogenic in nature, with only lytic phages being appropriate for phage treatment. This is due to the fact that lysogenic phages do not always result in lytic infection, leaving some bacteria alive with the phage genome inserted into their own. Additionally, lysogenic phages are capable of contributing to the transfer of antibiotic resistance and virulence genes across bacterial populations (Wagner and Waldor, 2002; Balcazar, 2014). Because of these factors, in addition to utilizing cocktails of phages to prevent the development of resistance, it is important that all phages to be used for treatment or prophylaxis in animal production be thoroughly tested to ensure purely lytic infections can occur with their use.

### Antibacterial Metals, Minerals, and Nanoparticles

Unlike phage, metals including Copper (Cu^2+^/Cu^+^), Zinc (Zn^2+^), and Silver (Ag^+^), and nonmetal elements, such as Iodine (I_2_), have been used in animal production for their broad-spectrum antibacterial activity and low generation of resistance (Aarestrup and Hasman, 2004; Murdoch and Lagan, 2013; Wang et al., 2016). Copper, zinc, and silver disrupt bacterial protein functions, generate reactive oxygen species, and cause damage to bacterial DNA (Rosen, 2002; Xiu et al., 2014). Although iodine’s antimicrobial activity is not well understood, there is indication that it works by reacting with unsaturated fatty acids in the lipid bilayer of the cell wall to cause leaks, as well as inactivating nuclear materials through coagulation (Murdoch and Lagan, 2013). As a result of their broad-spectrum antimicrobial activity, it was believed that generation of bacterial resistance to Cu^2+^, Zn^2+^, Ag^+^, and I_2_ should be low (Martínez-Abad et al., 2012; Murdoch and Lagan, 2013; Wang et al., 2016).

Copper and Zinc salts are commonly added to animal feeds in concentrations above dietary requirements because of their antimicrobial activity, which results in reduced infection and improved animal growth. Similarly, Zinc Oxide added to pig diets has been effective in reducing post-weaning diarrhea (Mazaheri Nezhad Fard et al., 2011; Pieper et al., 2012; Holman and Chénier, 2015; Vahjen et al., 2015). Additionally, copper has been determined to be an effective antimicrobial for udder washes, proving active against a panel of bacteria and yeasts associated with bovine mastitis (Reyes-Jara et al., 2016). The antimicrobial properties of copper and zinc when added to feed have created a selective pressure for bacteria that contain resistance to these heavy metals (Mazaheri Nezhad Fard et al., 2011). High inclusion rates alter the gut microbiome, however, many bacteria have developed resistance, with both zinc- and copper-resistant enterococci identified from the gut microbiome of pigs (Mazaheri Nezhad Fard et al., 2011; Pieper et al., 2012; Vahjen et al., 2015). Bacterial resistance genes to zinc and copper are located on mobile genetic elements, often plasmids, which are transferable between bacteria (Aarestrup and Hasman, 2004; Mazaheri Nezhad Fard et al., 2011; Richard et al., 2017).

**Figure 2.**
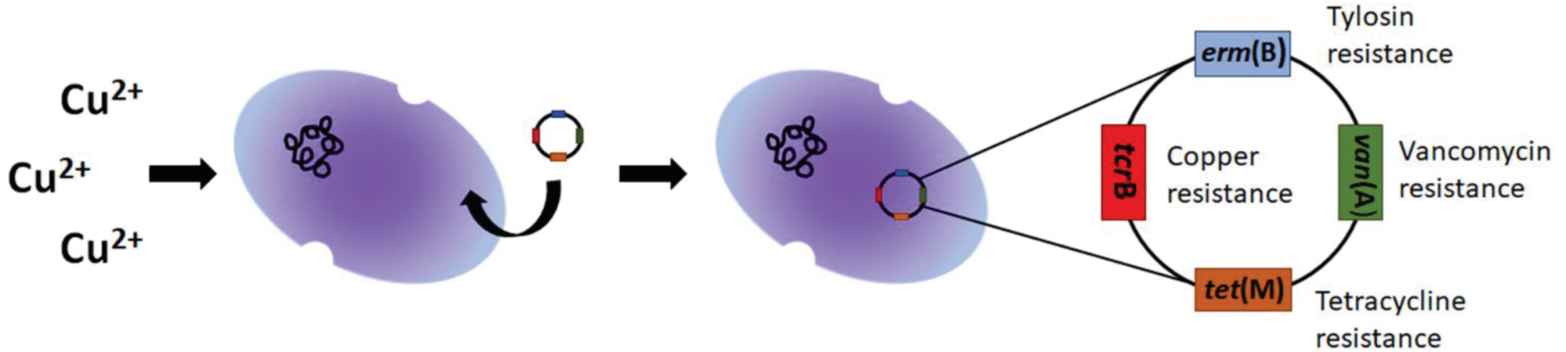
The selective pressure of copper results in the uptake of foreign plasmids by enterococci, conferring copper resistance genes as well as antibiotic resistance genes (coresistance).

More importantly, bacteria resistant to copper and zinc have indicated increased resistance to antibiotics, as an increased dose of dietary zinc oxide in weaned pigs increased tetracycline and sulfonamide resistance genes (Mazaheri Nezhad Fard et al., 2011; Vahjen et al., 2015). This increase in resistance is likely due to mechanisms of cross-resistance or coresistance: when microbes use the same resistance mechanism to defend against different antimicrobials such as an efflux pump, or when the genes responsible for resistance are linked closely and are transcribed or transferred together (El Behiry et al., 2012; Vahjen et al., 2015; Reyes-Jara et al., 2016). The genes associated with resistance to copper and zinc have been found on the same plasmids that contain antibiotic resistance genes, and the selective pressure of these metals can result in the sharing of antibiotic resistance among bacteria (Mazaheri Nezhad Fard et al., 2011; Yin et al., 2017), as depicted in Figure 2.

Silver and iodine have been used for many years as antimicrobial agents for wounds and external infections because of their broad-spectrum activity against bacteria and low development of resistance (Martínez-Abad et al., 2012; Murdoch and Lagan, 2013; Kalan et al., 2017). Iodine and silver are active antimicrobial ingredients used in both human and animal wound care products (Burks, 1998; Murdoch and Lagan, 2013; Kalan et al., 2017), and iodine has commonly been used as an udder wash (Tremblay et al., 2014).

Extracellular polymeric substances found in biofilms contain functional groups capable of binding metal ions, like silver, and protect against antimicrobial agents such as iodine (Kang et al., 2014; Tremblay et al., 2014; Xiu et al., 2014). Other defenses include mechanisms against oxidative stress, protein/DNA damage repair mechanisms, and metal efflux pumps (Gupta et al., 1999; Tremblay et al., 2014; Xiu et al., 2014). However, studies have shown that silver is capable of penetrating and killing biofilms (Heidari Zare et al., 2017; Kalan et al., 2017; Lemire et al., 2017) and multidrug-resistant pathogens (Kalan et al., 2017).

Recent research has indicated that silver resistance is due to the Sil operon that resides on plasmid pMG101 identified in *Salmonella enterica* serovar Typhimurium, which when transferred to *E. coli*, conferred silver resistance (Woods et al., 2009; Asiani et al., 2016). Plasmid pMG101 also contains resistance genes to a list of antibiotics, including ampicillin, chloramphenicol, tetracycline, streptomycin, and sulphonamide, suggesting that the transfer of pMG101 between bacteria under the selective pressure of silver may also result in sharing of other antibiotic resistance (Woods et al., 2009). However, incidence of silver resistance remains low (Woods et al., 2009; Kalan et al., 2017) which may indicate that plasmid pMG101 is restricted to particular species or is difficult to transfer or be maintained by other bacteria (Woods et al., 2009).

Up until 2013, no known generation of bacterial resistance to iodine had been identified, and studies looking at repeated iodine use over time did not indicate any increase in resistant bacteria (Murdoch and Lagan, 2013). Mastitis-associated bacteria have shown to produce biofilms to survive treatment with low concentrations of iodine (Tremblay et al., 2014). Using sublethal concentrations of nonoxinol-9 iodine complex on *Staphylococcus aureus* strains specific to mastitis resulted in the development of resistance, although the mechanisms of tolerance are unknown (El Behiry et al., 2012). Although other research has indicated cross-resistance of antibiotics with other biocides (El Behiry et al., 2012), currently there is no known cross-resistance with iodine and antibiotics (El Behiry et al., 2012; Murdoch and Lagan, 2013).

Given the challenges with feeding high dose metals, in particular, bacterial resistance and environmental effects of run-off, metal nanoparticles have gained attention as an alternative (Yin et al., 2017). Metal nanoparticles such as silver, copper oxide, and zinc oxide are of particular interest for their antimicrobial properties and suitability as feed additives (Beyth et al., 2015). Technological advances have decreased the cost of synthesizing nanoparticles and made their inclusion in livestock diets more feasible in recent years (Fondevila et al., 2009). The mode of action for antimicrobial metal nanoparticles is not completely elucidated; potential mechanisms include cell membrane disruption, generation of reactive oxygen species, and disruption of protein structure (Beyth et al., 2015). The increased surface area to volume ratio of smaller particles, as well as properties such as shape, can all contribute to an increased bactericidal activity compared to their corresponding metal ions (Gautam and Van Veggel, 2013; Rudramurthy et al., 2016). Zinc oxide nanoparticles have been demonstrated as effective bactericidal agents against antibiotic resistant *S. aureus* and *Staphylococcus epidermidis* (Ansari et al., 2012). Silver nanoparticles have also been shown to be effective against bacterial and fungal species, including some important pathogens (Rudramurthy et al., 2016).

While in many respects metal nanoparticles may be a promising tool, use of this technology could also generate bacterial resistance. Strain-specific minimum inhibitory concentrations of nanoparticles in *E. coli* and *S. aureus* have already been identified, demonstrating that varied resistance to nanoparticle antimicrobial mechanisms exist naturally in the bacterial population (Ruparelia et al., 2008). There is also the risk of accumulation of nanoparticles in livestock tissues particularly if these products are used over long time periods, and the implications for animal health and food safety are not yet completely understood (Fondevila et al., 2009; De Jong et al., 2013; Adeyemi and Faniyan, 2014). Prior to use, it will be necessary to determine if interaction between a specific nanoparticle and biological tissues results in undesired degradation by-products, inflammation, or oxidative stress (Gautam and Van Veggel, 2013; Rudramurthy et al., 2016). Metal nanoparticles may be able to confer similar or improved benefits as antibiotic alternatives in livestock; however, more work needs to be done to fully understand their antimicrobial mechanisms, and impacts on tissues and the environment before they can be readily used in livestock production systems.

### Organic Acids

Organic acids have been used in the food industry as preservatives and disinfectants for many years, and more recently have gained interest as feed additives for livestock (Ricke, 2003; Upadhaya et al., 2014). Some organic acids that have been tested include formic, acetic, sorbic, fumaric, lactic, propionic, citric, and benzoic acid. The proposed mode of action for organic acids involves conversion into their antibacterial forms in the gastrointestinal tract and diffusion into bacterial cells, decreasing cell internal pH (Ricke, 2003; Bearson et al., 1997).

Providing a mixture of organic acids to finisher pigs or broiler chicks has been shown to decrease *E. coli* counts and increase growth performance (Mohamed et al., 2014; Upadhaya et al., 2014). Similarly, the addition of formic or propionic acid in a *Salmonella* infection model decreased cecal *Salmonella* counts in chicks at 7, 14, and 21 days of age (McHan and Shotts, 2015). These effects on common pathogens suggest that organic acids could be a promising alternative to antibiotics; however, results are contradictory. In a different experiment, formic acid inclusion resulted in no effect on average daily gain of broiler chickens compared to a control diet, though changes in intestinal morphology were observed (Garcia et al., 2007). It is still unclear whether organic acids can improve growth performance and animal health across different livestock production settings, or if their efficacy is reliant on certain external factors. In stored feed the concentration of organic acids required to decrease pathogens depends on feed composition and pathogen status; for example, bacteria in stationary phase may be more resistant (Ricke, 2003). In vivo, their efficacy depends in part on factors such as pH, where low pH allows more undissociated acid to remain intact and functional (Baik et al., 1996). Protection via encapsulation may also improve efficacy by supplying acids to the intestine in their undissociated form and preventing absorption or metabolism before the products reach their desired location (Upadhaya et al., 2014).

Perhaps the most concerning drawback to the use of organic acids is their ability to induce acid tolerance responses in exposed bacteria. This tolerance response can result in the ability to withstand short-term exposure to pH as low as 3 (Baik et al., 1996). Over one generation, bacteria can increase tolerance to more extreme acid conditions (Ricke, 2003; Bearson et al., 1997). Bacterial species, such as *Salmonella*, naturally encounter low pH as well as short-chain fatty acids as part of their transit of the gastrointestinal tract, and can cope with these stressors using RNA polymerase, sigma S dependant systems (Baik et al., 1996). This acid stress response can increase pathogen survival in the stomach or in phagosomes, leading to increased virulence in both *Salmonella* and pathogenic *E. coli* (Ricke, 2003; Bearson et al., 1997). Acid tolerance has also been shown to increase shedding of *E. coli* O157:H7 in calves and mice (Price et al., 2000). Acid adaptation can also improve bacterial resistance to heat, salt, and H_2_O_2_, which could have serious implications for food safety and preservation (Bearson et al., 1997). Increased resistance to these other stressors may also lead to bacterial resistance to other antimicrobial alternatives such as metal ions or nanoparticles, further decreasing the number of tools available to help maintain animal health.

### Essential Oils

Extracted oils from the roots, seeds, leaves, bark, flowers, and fruits of plants contain complex mixtures of phenolic compounds known for their antimicrobial, anti-inflammatory, and antioxidants activities (Ayseli and Ipek Ayseli, 2016). The bioactive components in essential oils can modify both bacterial and host cellular functions by interacting with cell wall components and lipid membranes, which in the right conditions can lead to cell death (Omonijo et al., 2017). A major concern, as with antibiotics, is that over time bacteria may adapt and become resistant to the active phenolic components.

Studies have evaluated the antibacterial activity of essential oils and purified phytogenic compounds on both pathogenic and gut commensal bacteria. Pathogenic bacteria are sensitive to an array of essential oils at concentrations ranging from 0.02 to 0.7 g/L (Yang et al., 2015). Antimicrobial activity was assessed in 28 different essential oils by disk diffusion method on pathogenic *S. enterica* and on beneficial *Lactobacillus plantarum*. From the evaluation, essential oils from oranges had the best selective antibacterial activity against pathogenic bacteria with diminished activity on beneficial species (Ambrosio et al., 2017). Essential oils have been shown to inhibit multidrug-resistant bacteria independently of their antibiotic resistance profile (Becerril et al., 2012). Antifungal properties have also been documented against multiple strains of *Candida albicans* isolated from cattle with mastitis. Rosemary terpenes are suggested to act together on *C. albicans* by disrupting cellular integrity, respiration, ion transport, and membrane permeability (Ksouri et al., 2017). Similarly, citrus essential oils are suspected to disrupt the cell membrane due to the less abundant compounds working synergistically rather than one dominant compound such as thymol and carvacrol found in select herbal essential oils (Ambrosio et al., 2017). In this light, the antimicrobial mode of action of essential oils may be specific to one compound or the result of many.

The effectiveness of essential oils against common pathogens, *E. coli* O157:H7 and *S. enterica*, is dependent on the concentration of active phenolic compounds (Friedman et al., 2017). A cause of concern comes from an outbreak of *S. enterica* infections traced back to contaminated basil leaves. The subinhibitory concentrations of basil permitted *S. enterica* to develop resistance to its active component linalool (Kisluk et al., 2013). Bacterial resistance mechanisms toward essential oils include selective membrane permeability, regulated efflux/influx and chemotaxis-controlled motility (Kalily et al., 2016). Linalool-associated adaptations, although protective in vitro, may have a significant fitness cost on the adapted bacteria in a challenging environment (Kalily et al., 2017). Bacteria-associated adaptations complicate the use of essential oils and long-term studies are needed to understand whether the adapted resistance is a transmissible function.

Phytogenic compounds have good potential as an alternative to antibiotics in animal production, both as growth promoters and as treatment for bacterial infections (Omonijo et al., 2017; Reyer et al., 2017). Concentrations of the active components must be tested in vivo to determine whether an effective dose can be reasonably achieved. Additionally, their lipophilic nature may limit delivery to enteric pathogens, but again, microencapsulation for targeted release can help (Yang et al., 2015). A new strategy to combine essential oils with either disruptive metals, antibiotics, and/or nanotechnologies has gained attention to effectively combat multiresistant strains of bacteria and reduce bacterial resistance (Kwiatkowski et al., 2017; Low et al., 2017; Omonijo et al., 2017). The synergistic effect of phenolic compounds in combination with other environmental challenging applications may be a safer and more effective approach to address growing concerns of bacterial resistance.

### Microbial Approaches

Direct-fed microbials, or probiotics, have been evaluated as alternatives to antimicrobial growth promoters in livestock production. The effectiveness of direct-fed microbials as growth promoters and therapeutic antimicrobials is highly variable (McAllister et al., 2011), but positive effects, such as improving feed efficiency, weight gain, nutrient digestibility, intestinal morphology, and reducing potential pathogens and diarrhea occurrence have been reported in livestock species (Salim et al., 2013; Hou et al., 2015; Lei et al., 2015; Uyeno et al., 2015).

The strains selected for use as probiotics must be evaluated for the presence of antimicrobial resistance genes that could potentially be transferred to pathogenic bacteria in the gut. Antibiotic susceptibility in 46 *Lactobacillus* strains obtained from the human gut and dairy products was evaluated by disc-diffusion method, and all strains had shown resistance to a group of 14 antibiotics (Charteris et al., 1998). One strain of *Lactobacillus reuteri*, a probiotic candidate that can reduce *E. coli* and *Salmonella pullorum* growth in vitro, showed resistance to several antibiotics, including tetracycline, chloramphenicol, vancomycin, streptomycin, bacitracin, and penicillin G (Zhang et al., 2012). Identification and removal of resistance determinants, creating mutants with similar probiotic capacities, can potentially be used as a strategy to overcome the risk of horizontal gene transfer from probiotics to pathogens (Rosander et al., 2008).

Several direct-fed microbial strains produce short-chain fatty acids, which can reduce gut pH and inhibit pathogen growth (Kamada et al., 2013). However, in vitro studies found that *Listeria monocytogenes* can develop tolerability to acidic conditions, which is associated with increased resistance to other stressors and increased virulence (O’Driscoll et al., 1996; Conte et al., 2000), and as discussed above.

A promising microbial approach that might replace or complement antibiotic treatments is the use of purified bacteriocins or bacteriocin-producing microorganisms (Cavera et al., 2015). Bacteriocins are peptides ribosomally synthesized by bacteria and archaea (Riley and Wertz, 2002; Cotter et al., 2005) that vary in size, structure, mechanism of action, antimicrobial potency, immunity mechanisms, target cell receptors (Gillor et al., 2008), and bactericidal spectrum (Cotter et al., 2005). Bacteriocins can facilitate the dominance of a producer in a competitive environment (Dawid et al., 2007), regulate gut microbiota (Bhardwaj et al., 2010), and inhibit pathogen growth, without affecting others members of the microbial community (Dabour et al., 2009). The mechanisms by which bacteriocins exert their bactericidal and bacteriostatic effects include cell wall and plasma membrane disruption, impairment of protein synthesis, interference with DNA replication and transcription, and induction of cell autolysis (Cavera et al., 2015; Ahmad et al., 2017).

Bacteriocins can be used to treat infectious diseases such as mastitis caused by *Streptococcus dysgalactiae* in lactating cows (Ryan et al., 1998), to preserve food products, and to promote the establishment of probiotic strains (Cotter et al., 2005; Cotter et al., 2013; Martinez et al., 2016). However, innate and/or acquired resistance to bacteriocins are frequently reported (Ahmad et al., 2017). Identified mechanisms of bacteriocin resistance in Gram positive bacteria include cell wall modifications and alteration of the cell membrane composition, which affect membrane fluidity and electrical charges, therefore impairing bacteriocin ability to bind to bacterial cells. These mechanisms are similar to those of resistance to antibiotics, and this similarity raises concern regarding the development of cross-resistance (Zhou et al., 2014). Bacteriocin-resistant *L. monocytogenes* downregulate the expression of mannose phosphotransferase system, impairing the binding of bacteriocins to the cell membrane; but this resistance is accompanied by a reduction in pathogen growth rate compared to the sensitive strain (Masias et al., 2017), indicating that the development of resistance to bacteriocins may increase energy costs and compromise fitness of resistant strains (Martinez et al., 2016). Some bacteriocins have been engineered for improved efficacy and stability (Cavera et al., 2015), and their use as therapeutic agents is a rapidly developing area of research (Lohans and Vederas, 2012). However, there are currently only a few commercially bacteriocin-based products available for veterinary use (Martinez et al., 2016).

## Conclusion

**Figure 3.**
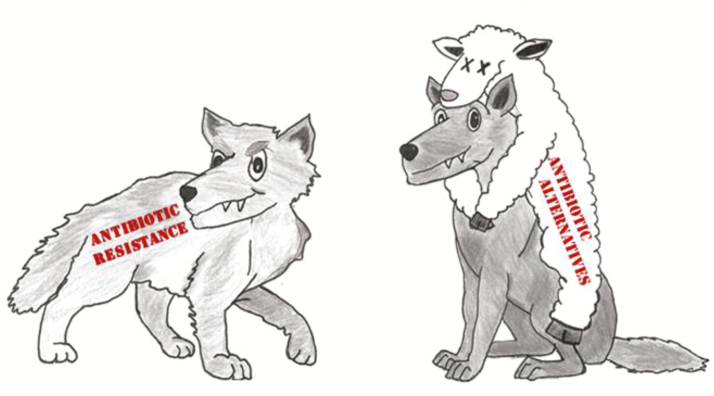
Antibiotic alternatives may be a danger in disguise.


It is clear that there is substantial effort going into the development of antibiotic alternatives to support healthy and efficient animal production. As we move forward with these technologies, it is important to keep resistance mechanisms in mind so that these technologies can be sustained. Most importantly, some of these antibiotic alternatives, such as zinc oxide, can clearly contribute to increased antibiotic resistance and should therefore be avoided. For other antibiotic alternatives, such as phage and bacteriocins, the potential contribution to antibiotic resistance is less clear, but should be considered. The idea of using more specific antibiotic alternative therapies, such as bacteriophage and bacteriocins, is enticing, as they do not affect commensal microbes. Furthermore, new phage will always be available as a result of the constant arms race between bacteria and bacteriophage. Ultimately, any strategy used must be economically viable, but we must avoid complacency and ensure that we are not replacing a wolf (antibiotic resistance) with a wolf in sheep’s clothing (Figure 3).

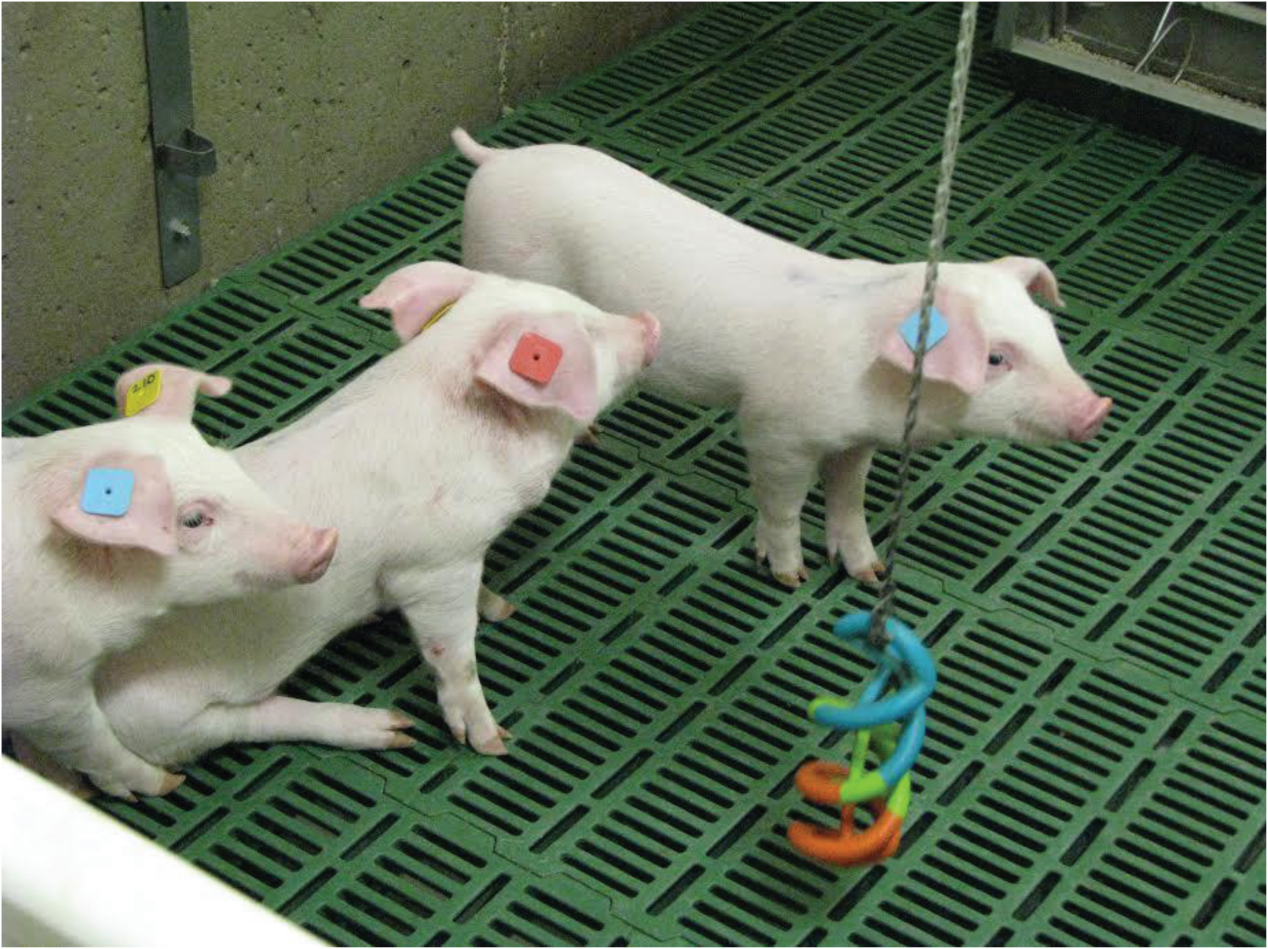


Pig image: many antimicrobial alternatives target the early post-weaning piglet.


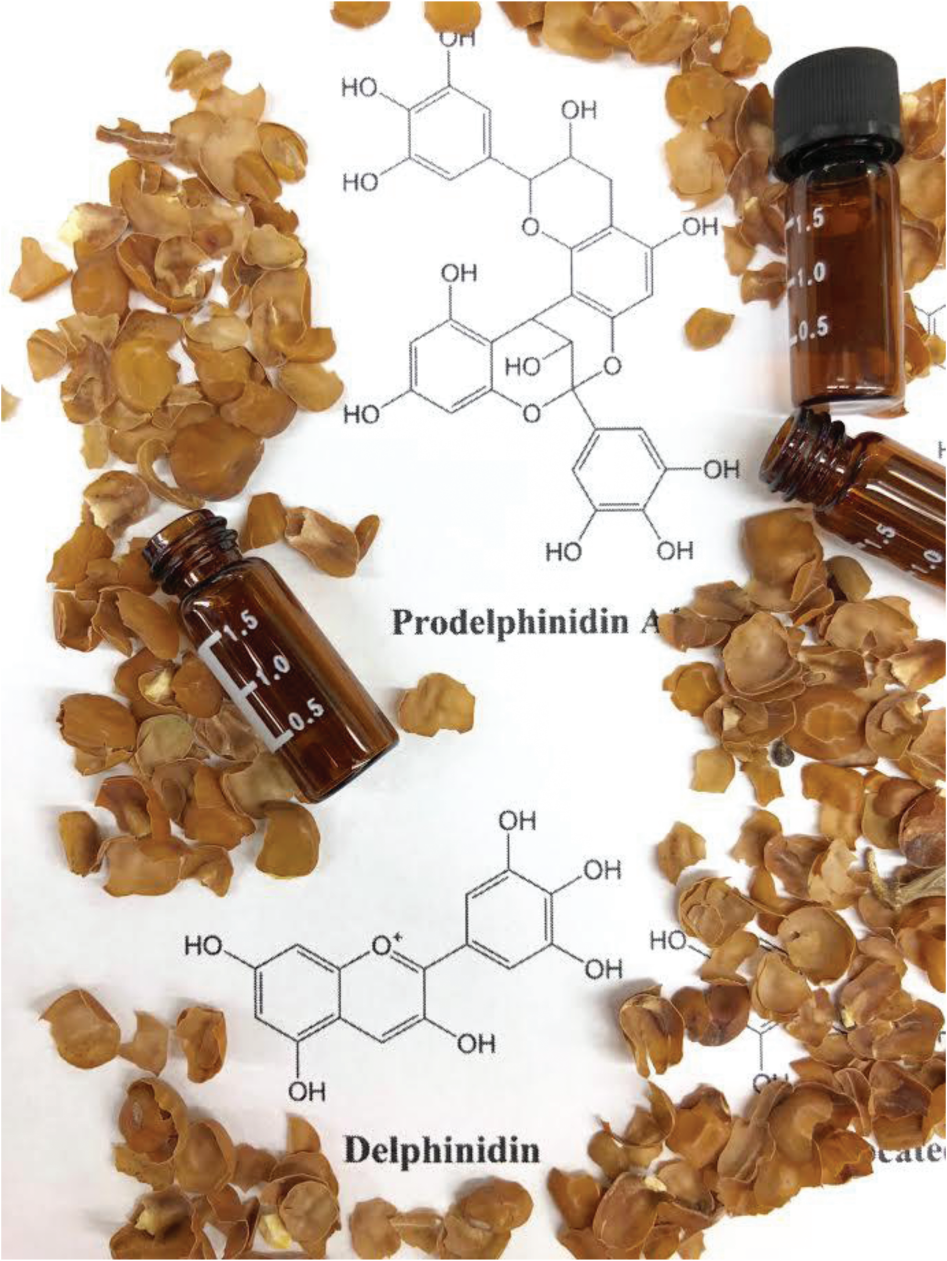


Polyphenols: polyphenolic compounds found in plant products, such as these pea seed coats, have strong antibacterial activity.

## About the Authors

**Dr. Benjamin Willing** is an assistant professor at the University of Alberta and Canada Research Chair in the Microbiology of Nutrigenomics. He completed a bachelor’s degree in Animal Science at University of British Columbia after which he completed his Ph.D. at the University of Saskatchewan. He was recently recognized with an International Life Sciences Institute Future Leader Award. His current research focuses on nutrient–microbe–host interactions, and he uses antibiotic and germ-free animal models to study how microbes regulate host physiology in regards to metabolism and disease resistance.

**Deanna Pepin** is a graduate student at the University of Alberta in the Department of Agricultural, Food and Nutritional Science under the supervision of Dr. Willing. The focus of her current research is how iodinated water impacts the development of the gastrointestinal microbiota, *Salmonella* infection resistance, and immune response in broiler production. She has also done work investigating the minimum inhibitory concentration of different metals on multidrug-resistant pathogens.

**Camila Marcolla** is a Ph.D. student in the Department of Agricultural, Food and Nutritional Science at the University of Alberta, under the supervision of Dr. Benjamin Willing. Her project is focused on characterizing the chicken gut microbiome, creating a culture collection of microbes, and developing strategies to manipulate the chicken gut microbiome to facilitate the establishment of farming systems that are less dependent on antibiotic use.

**Andrew Forgie** is a Ph.D. student in the Department of Agricultural, Food and Nutritional Sciences at the University of Alberta, under the supervision of Dr. Benjamin Willing. His research focuses on the impact of diet, specifically phytochemicals, on host health with particular interest in their effects on the gut microbiome and intestinal integrity.

**Natalie Diether** is a Ph.D. student in the Department of Agricultural, Food, and Nutritional Science at the University of Alberta. Supervised by Dr. Ben Willing and Dr. Paul Stothard, her current research is focused on exploring the interaction between feed, gut microbiome, and host response in pigs.

**Benjamin Bourrie** is a Ph.D. candidate in the Department of Agricultural, Food, and Nutritional Science at the University of Alberta. As a Walsh Fellow, he is cosupervised by Dr. Benjamin Willing at the University of Alberta and Dr. Paul Cotter at Teagasc in Fermoy, Ireland. The focus of his current research is the impact of kefir on obesity, cholesterol metabolism, and nonalcoholic fatty liver disease. His past research includes the isolation and characterization of bacteriophages for their potential use in phage therapy.

## References

[CIT0001] Aarestrup F. M., and Hasman H. 2004 Susceptibility of different bacterial species isolated from food animals to copper sulphate, zinc chloride and antimicrobial substances used for disinfection. Vet. Microbiol. 100:83–89. doi:10.1016/j.vetmic.2004.01.013 15135516 10.1016/j.vetmic.2004.01.013

[CIT0002] Adeyemi O. S., and Faniyan T. O. 2014 Antioxidant status of rats administered silver nanoparticles orally. J. Taibah Univ. Med. Sci. 9:182–186. doi:10.1016/j.jtumed.2014.03.002

[CIT0003] Ahmad V., Khan M. S., Jamal Q. M. S., Alzohairy M. A., Al Karaawi M. A., and Siddiqui M. U. 2017 Antimicrobial potential of bacteriocins: in therapy, agriculture and food preservation. Int. J. Antimicrob. Agents. 49:1–11. doi:10.1016/j.ijantimicag.2016.08.016 27773497 10.1016/j.ijantimicag.2016.08.016

[CIT0004] Ambrosio C. M. S., de Alencar S. M., de Sousa R. L. M., Moreno A. M., and Da Gloria E. M. 2017 Antimicrobial activity of several essential oils on pathogenic and beneficial bacteria. Ind. Crops Prod. 97:128–136. doi:10.1016/j.indcrop.2016.11.045

[CIT0005] Ansari M. A., Khan H. M., Khan A. A., Sultan A., and Azam A. 2012 Characterization of clinical strains of MSSA, MRSA and MRSE isolated from skin and soft tissue infections and the antibacterial activity of ZnO nanoparticles. World J. Microbiol. Biotechnol. 28:1605–1613. doi:10.1007/s11274-011-0966-1 22805942 10.1007/s11274-011-0966-1

[CIT0006] Asiani K. R., Williams H., Bird L., Jenner M., Searle M. S., Hobman J. L., Scott D. J., and Soultanas P. 2016 Sile is an intrinsically disordered periplasmic “molecular sponge” involved in bacterial silver resistance. Mol. Microbiol. 101:731–742. doi:10.1111/mmi.13399 27085056 10.1111/mmi.13399 PMC5008109

[CIT0007] Ayseli M. T., and Ipek Ayseli Y. 2016 Flavors of the future: Health benefits of flavor precursors and volatile compounds in plant foods. Trends Food Sci. Technol. 48:69–77. doi:10.1016/j.tifs.2015.11.005

[CIT0008] Bach S. J., Johnson R. P., Stanford K., and Mcallister T. A. 2009 Bacteriophages reduce *Escherichia coli* O157 : H7 levels in experimentally inoculated sheep. Can. J. Anim. Sci. 89:285–293. doi:10.4141/CJAS08083

[CIT0100] Aminov R. I., and Mackie R. I. 2007 Evolution and ecology of antibiotic resistance genes. FEMS Microbiol. Lett. 271:147–161. doi:10.1111/j.1574-6968.2007.00757.x 17490428 10.1111/j.1574-6968.2007.00757.x

[CIT0009] Baik H. S., Bearson S., Dunbar S., and Foster J. W. 1996 The acid tolerance response of *Salmonella typhimurium* provides protection against organic acids. Microbiology. 142:3195–3200. doi:10.1099/13500872-142-11-3195 8969516 10.1099/13500872-142-11-3195

[CIT0010] Balcazar J. L 2014 Bacteriophages as vehicles for antibiotic resistance genes in the environment. Plos Pathog. 10:e1004219. doi:10.1371/journal.ppat.1004219 25078987 10.1371/journal.ppat.1004219 PMC4117541

[CIT0011] Bardina C., Spricigo D. A., Cortés P., and Llagostera M. 2012 Significance of the bacteriophage treatment schedule in reducing *Salmonella* colonization of poultry. Appl. Environ. Microbiol. 78:6600–6607. doi:10.1128/AEM.01257-12 22773654 10.1128/AEM.01257-12 PMC3426709

[CIT0012] Bearson S., Bearson B., and Foster J. W. 1997 Acid stress responses in enterobacteria. FEMS Microbiol. Lett. 147:173–180. doi:10.1111/j.1574–6968.1997.tb10238.x 9119190 10.1111/j.1574-6968.1997.tb10238.x

[CIT0013] Becerril R., Nerín C., and Gómez-Lus R. 2012 Evaluation of bacterial resistance to essential oils and antibiotics after exposure to oregano and cinnamon essential oils. Foodborne Pathog. Dis. 9:699–705. doi:10.1089/fpd.2011.1097 22827568 10.1089/fpd.2011.1097

[CIT0014] Beyth N., Houri-Haddad Y., Domb A., Khan W., and Hazan R. 2015 Alternative antimicrobial approach: nano-antimicrobial materials. Evid. Based. Complement. Alternat. Med. 2015:246012. doi:10.1155/2015/246012 25861355 10.1155/2015/246012 PMC4378595

[CIT0015] Bhardwaj A., Gupta H., Kapila S., Kaur G., Vij S., and Malik R. K. 2010 Safety assessment and evaluation of probiotic potential of bacteriocinogenic enterococcus faecium KH 24 strain under in vitro and in vivo conditions. Int. J. Food Microbiol. 141:156–164. doi:10.1016/j.ijfoodmicro.2010.05.001 20570005 10.1016/j.ijfoodmicro.2010.05.001

[CIT0016] Borie C., Albala I., Sánchez P., Sánchez M. L., Ramírez S., Navarro C., Morales M. A., Retamales A. J., and Robeson J. 2008 Bacteriophage treatment reduces *Salmonella* colonization of infected chickens. Avian Dis. 52:64–67. doi:10.1637/8091-082007-Reg 18459298 10.1637/8091-082007-Reg

[CIT0017] Burks R. I 1998 Povidone-iodine solution in wound treatment. Phys. Ther. 78:212–218. PMID: 9474112. 9474112 10.1093/ptj/78.2.212

[CIT0018] Carrillo C. L., Atterbury R. J., Dillon E., A Scott I. F. Connerton, and Connerton P. L. 2005 Bacteriophage therapy to reduce *Campylobacter jejuni* colonization of broiler chickens bacteriophage therapy to reduce *Campylobacter jejuni* colonization of broiler chickens. Appl. Environ. Microbiol. 71:6554–6563. doi:10.1128/AEM.71.11.6554 16269681 10.1128/AEM.71.11.6554-6563.2005 PMC1287621

[CIT0019] Cavera V. L., Arthur T. D., Kashtanov D., and Chikindas M. L. 2015 Bacteriocins and their position in the next wave of conventional antibiotics. Int. J. Antimicrob. Agents. 46:494–501. doi:10.1016/j.ijantimicag.2015.07.011 26341839 10.1016/j.ijantimicag.2015.07.011

[CIT0020] Charteris W. P., Kelly P. M., Morelli L., and Collins4 A. J. K. 1998 Antibiotic susceptibility of potentially probiotic lactobacillus species. J. Food Prot. 6:636–643. doi:10.4315/0362-028X-61.12.1636. 10.4315/0362-028x-61.12.1636 9874341

[CIT0021] Conte M., Petrone G., Di Biase A., Ammendolia M., Superti F., and Seganti L. 2000 Acid tolerance in *Listeria monocytogenes* influences invasiveness of enterocyte-like cells and macrophage-like cells. Microb. Pathog. 29:137–144. doi:10.1006/mpat.2000.0379 10968945 10.1006/mpat.2000.0379

[CIT0022] Cotter P. D., Hill C., and Ross R. P. 2005 Bacteriocins: developing innate immunity for food. Nat. Rev. Microbiol. 3:777–788. doi:10.1038/nrmicro1273 16205711 10.1038/nrmicro1273

[CIT0023] Cotter P. D., Ross R. P., and Hill C. 2013 Bacteriocins-a viable alternative to antibiotics?Nat. Rev. Microbiol. doi:10.1038/nrmicro2937 10.1038/nrmicro2937 23268227

[CIT0024] Cryz S. J. Jr, Pitt T. L., Fürer E., and Germanier R. 1984 Role of lipopolysaccharide in virulence of *Pseudomonas aeruginosa*. Infect. Immun. 44:508–513. PMCID: PMC263549. 6425224 10.1128/iai.44.2.508-513.1984 PMC263549

[CIT0025] Dabour N., Zihler A., Kheadr E., Lacroix C., and Fliss I. 2009 In vivo study on the effectiveness of pediocin PA-1 and *Pediococcus acidilactici* UL5 at inhibiting *Listeria monocytogenes*. Int. J. Food Microbiol. 133:225–233. doi:10.1016/j.ijfoodmicro.2009.05.005 19541383 10.1016/j.ijfoodmicro.2009.05.005

[CIT0026] Dawid S., Roche A. M., and Weiser J. N. 2007 The blp bacteriocins of *Streptococcus pneumoniae* mediate intraspecies competition both in vitro and in vivo. Infect. Immun. 75:443–451. doi:10.1128/IAI.01775-05 17074857 10.1128/IAI.01775-05 PMC1828380

[CIT0027] De Jong W. H., Van Der Ven L. T. M., Sleijffers A., Park M. V. D. Z., Jansen E. H. J. M., Van Loveren H., and Vandebriel R. J. 2013 Biomaterials Systemic and immunotoxicity of silver nanoparticles in an intravenous 28 days repeated dose toxicity study in rats. Biomaterials. 34:8333–8343. doi:10.1016/j.biomaterials.2013.06.048 23886731 10.1016/j.biomaterials.2013.06.048

[CIT0028] El Behiry A., Schlenker G., Szabo I., and Roesler U. 2012 In vitro susceptibility of *Staphylococcus aureus* strains isolated from cows with subclinical mastitis to different antimicrobial agents. J. Vet. Sci. 13:153–161. doi:10.4142/jvs.2012.13.2.153 22705737 10.4142/jvs.2012.13.2.153 PMC3386340

[CIT0029] Fondevila M., Herrer R., Casallas M. C., Abecia L., and Ducha J. J. 2009 Silver nanoparticles as a potential antimicrobial additive for weaned pigs. Anim. Feed Sci. Technol. 150:259–269. doi:10.1016/j.anifeedsci.2008.09.003

[CIT0030] Friedman M., Levin C. E., and Henika P. R. 2017 Addition of phytochemical-rich plant extracts mitigate the antimicrobial activity of essential oil/wine mixtures against *Escherichia coli* O157:H7 but not against *Salmonella enterica*. Food Control. 73:562–565. doi:10.1016/j.foodcont.2016.09.002

[CIT0031] Garcia V., Catala-Gregori P., Hernandez F., Megıas M. D., and Madrid J. 2007 Effect of formic acid and plant extracts on growth, nutrient digestibility, intestine mucosa morphology, and meat yield of broilers JAPR : research report. J. Appl. Poult. Res. 16:555–562. doi:10.3382/japr.2006-00116

[CIT0032] Gautam A., and Van Veggel F. C. J. M. 2013 Synthesis of nanoparticles, their biocompatibility, and toxicity behavior for biomedical applications. J. Mater. Chem. B. 1:5186–5200. doi:10.1039/c3tb20738b 10.1039/c3tb20738b 32263325

[CIT0033] Gillor O., Etzion A., and Riley M. A. 2008 The dual role of bacteriocins as anti- and probiotics. Appl. Microbiol. Biotechnol. 81:591–606. doi:10.1007/s00253-008-1726-5 18853155 10.1007/s00253-008-1726-5 PMC2670069

[CIT0034] Gresse R., Chaucheyras-Durand F., Fleury M. A., Van de Wiele T., Forano E., and Blanquet-Diot S. 2017 Gut microbiota dysbiosis in postweaning piglets: understanding the keys to health. Trends Microbiol. 25:851–873. doi:10.1016/j.tim.2017.05.004 28602521 10.1016/j.tim.2017.05.004

[CIT0035] Gupta A., Matsui K., Lo J. F., and Silver S. 1999 Molecular basis for resistance to silver cations in *Salmonella*. Nat. Med. 5:183–188. doi:10.1038/5545 9930866 10.1038/5545

[CIT0036] Heidari Zare H., Juhart V., Vass A., Franz G., and Jocham D. 2017 Efficacy of silver/hydrophilic poly(*p* -xylylene) on preventing bacterial growth and biofilm formation in urinary catheters. Biointerphases. 12:11001. doi:10.1116/1.4974197 10.1116/1.4974197 28100054

[CIT0037] Holman D. B., and Chénier M. R. 2015 Antimicrobial use in swine production and its effect on the swine gut microbiota and antimicrobial resistance. Can. J. Microbiol. 61:785–798. doi:10.1139/cjm-2015-0239 26414105 10.1139/cjm-2015-0239

[CIT0038] Hou C., Zeng X., Yang F., Liu H., and Qiao S. 2015 Study and use of the probiotic *Lactobacillus reuteri* in pigs: a review. J. Anim. Sci. Biotechnol. 6:14. doi:10.1186/s40104-015-0014-3 25954504 10.1186/s40104-015-0014-3 PMC4423586

[CIT0039] Huff W. E., Huff G. R., Rath N. C., Balog J. M., and Donoghue A. M. 2002 Prevention of *Escherichia coli* infection in broiler chickens with a bacteriophage aerosol spray. Poult. Sci. 81:1486–1491. doi:10.1093/ps/81.10.1486 12412913 10.1093/ps/81.10.1486

[CIT0040] Huff W. E., Huff G. R., Rath N. C., and Donoghue A. M. 2006 Evaluation of the influence of bacteriophage titer on the treatment of colibacillosis in broiler chickens. Poult. Sci. 85:1373–1377. doi:10.1093/ps/85.8.1373 16903466 10.1093/ps/85.8.1373

[CIT0041] Kalan L. R., Pepin D. M., Ul-Haq I., Miller S. B., Hay M. E., and Precht R. J. 2017 Targeting biofilms of multidrug-resistant bacteria with silver oxynitrate. Int. J. Antimicrob. Agents. 49:719–726. doi:10.1016/j.ijantimicag.2017.01.019 28390963 10.1016/j.ijantimicag.2017.01.019

[CIT0042] Kalily E., Hollander A., Korin B., Cymerman I., and Yaron S. 2016 Mechanisms of resistance to linalool in *Salmonella* senftenberg and their role in survival on basil. Environ. Microbiol. 18:3673–3688. doi:10.1111/1462-2920.13268 26914987 10.1111/1462-2920.13268

[CIT0043] Kalily E., Hollander A., Korin B., Cymerman I., and Yaron S. 2017 *Salmonella* Senftenberg adaptation to linalool and its association with antibiotic resistance and environmental persistence. Appl. Environ. Microbiol. 83:AEM.03398-16. doi:10.1128/AEM.03398-16 10.1128/AEM.03398-16 PMC5411494 28258149

[CIT0044] Kamada N., Chen G. Y., Inohara N., and Núñez G. 2013 Control of pathogens and pathobionts by the gut microbiota. Nat. Immunol. 14:685–690. doi:10.1038/ni.2608 23778796 10.1038/ni.2608 PMC4083503

[CIT0045] Kang F., Alvarez P. J., and Zhu D. 2014 Microbial extracellular polymeric substances reduce Ag ^+^ to silver nanoparticles and antagonize bactericidal activity. Environ. Sci. Technol. 48:316–322. doi:10.1021/es403796x 24328348 10.1021/es403796x

[CIT0046] Kisluk G., Kalily E., and Yaron S. 2013 Resistance to essential oils affects survival of *Salmonella enterica* serovars in growing and harvested basil. Environ. Microbiol. 15:2787–2798. doi:10.1111/1462-2920.12139 23648052 10.1111/1462-2920.12139

[CIT0047] Ksouri S., Djebir S., Bentorki A. A., Gouri A., Hadef Y., and Benakhla A. 2017 Antifungal activity of essential oils extract from origanum floribundum munby, *Rosmarinus officinalis* L. and *Thymus ciliatus* Desf. against *Candida albicans* isolated from bovine clinical mastitis. J. Mycol. Med. 27:245–249. doi:10.1016/j.mycmed.2017.03.004 28454927 10.1016/j.mycmed.2017.03.004

[CIT0048] Kwiatkowski P., Mnichowska-Polanowska M., Pruss A., Masiuk H., Dzięcioł M., Giedrys-Kalemba S., and Sienkiewicz M. 2017 The effect of fennel essential oil in combination with antibiotics on *Staphylococcus aureus* strains isolated from carriers. Burns. 43:1544–1551. doi:10.1016/j.burns.2017.04.014 28917968 10.1016/j.burns.2017.04.014

[CIT0049] Labrie S. J., Samson J. E., and Moineau S. 2010 Bacteriophage resistance mechanisms. Nat. Rev. Microbiol. 8:317–327. doi:10.1038/nrmicro2315 20348932 10.1038/nrmicro2315

[CIT0050] Lei X., Piao X., Ru Y., Zhang H., Péron A., and Zhang H. 2015 Effect of bacillus amyloliquefaciens-based direct-fed microbial on performance, nutrient utilization, intestinal morphology and cecal microflora in broiler chickens. Asian-Australas. J. Anim. Sci. 28:239–246. doi:10.5713/ajas.14.0330 25557820 10.5713/ajas.14.0330 PMC4283169

[CIT0051] Lemire J. A., Kalan L., Gugala N., Bradu A., and Turner R. J. 2017 Silver oxynitrate–an efficacious compound for the prevention and eradication of dual-species biofilms. Biofouling. 33:460–469. doi:10.1080/08927014.2017.1322586 28521545 10.1080/08927014.2017.1322586

[CIT0052] Lohans C. T., and Vederas J. C. 2012 Development of class iia bacteriocins as therapeutic agents. Int. J. Microbiol. 2012:386410. doi:10.1155/2012/386410 22187559 10.1155/2012/386410 PMC3236453

[CIT0053] Low W. L., Kenward K., Britland S. T., Amin M. C., and Martin C. 2017 Essential oils and metal ions as alternative antimicrobial agents: a focus on tea tree oil and silver. Int. Wound J. 14:369–384. doi:10.1111/iwj.12611 27146784 10.1111/iwj.12611 PMC7949732

[CIT0054] Martinez B., Rodriguez A., and Suarez E. 2016 Antimicrobial peptides produces by bacteria: the bacteriocins. In: T. G. Villa and M. Vinas, editors. New weapons to control bacterial growth. Switzerland: Springer International Publishing; p. 15–37. doi:10.1007/978-3-319-28368-5

[CIT0055] Martínez-Abad A., Sánchez G., Lagaron J. M., and Ocio M. J. 2012 On the different growth conditions affecting silver antimicrobial efficacy on *Listeria monocytogenes* and *Salmonella enterica*. Int. J. Food Microbiol. 158:147–154. doi:10.1016/j.ijfoodmicro.2012.07.010 22835228 10.1016/j.ijfoodmicro.2012.07.010

[CIT0056] Masias E., Dupuy F. G., da Silva Sanches P. R., Farizano J. V., Cilli E., Bellomio A., Saavedra L., and Minahk C. 2017 Impairment of the class IIa bacteriocin receptor function and membrane structural changes are associated to enterocin CRL35 high resistance in *Listeria monocytogenes*. Biochim. Biophys. Acta - Gen. Subj. 1861:1770–1776. doi:10.1016/j.bbagen.2017.03.014 28323072 10.1016/j.bbagen.2017.03.014

[CIT0057] Mazaheri Nezhad Fard R., Heuzenroeder M. W., and Barton M. D. 2011 Antimicrobial and heavy metal resistance in commensal enterococci isolated from pigs. Vet. Microbiol. 148:276–282. doi:10.1016/j.vetmic.2010.09.002 20951513 10.1016/j.vetmic.2010.09.002

[CIT0058] McAllister T. A., Beauchemin K. A., Alazzeh A. Y., Baah J., Teather R. M., and Stanford K. 2011 Review: the use of direct fed microbials to mitigate pathogens and enhance production in cattle. Can. J. Anim. Sci. 91:193–211. doi:10.4141/cjas10047

[CIT0059] McHan F., and Shotts E. B. 2015 Effect of feeding selected short-chain fatty acids on the in vivo attachment of *Salmonella typhimurium* in chick ceca. Avian Dis. 36:139–142. PMID: 1567301 1567301

[CIT0060] Mohamed M. A., El-Daly E. F., Abd El-Azeem N. A., Youssef A. W., and Hassan H. M. A. 2014 Growth performance and histological changes in ileum and immune related organs of broilers fed organic acids or antibiotic growth promoter. Int. J. Poult. Sci. 13:602–610. doi:10.3923/ijps.2014.602.610

[CIT0061] Murdoch R., and Lagan K. M. 2013 The role of povidone and cadexomer iodine in the management of acute and chronic wounds. Phys. Ther. Rev. 18:207–216. doi:10.1179/1743288X13Y.0000000082

[CIT0062] O’Driscoll B., Gahan C. G., and Hill C. 1996 Adaptive acid tolerance response in *Listeria monocytogenes*: isolation of an acid-tolerant mutant which demonstrates increased virulence. Appl. Environ. Microbiol. 62:1693–1698. PMCID: PMC167944. 8633868 10.1128/aem.62.5.1693-1698.1996 PMC167944

[CIT0063] Omonijo F. A., Ni L., Gong J., Wang Q., Lahaye L., and Yang C. 2017 Essential oils as alternatives to antibiotics in swine production. Anim. Nutr. 1–11. doi:10.1016/J.ANINU.2017.09.001 30140752 10.1016/j.aninu.2017.09.001 PMC6104524

[CIT0064] Pieper R., Vahjen W., Neumann K., Van Kessel A. G., and Zentek J. 2012 Dose-dependent effects of dietary zinc oxide on bacterial communities and metabolic profiles in the ileum of weaned pigs. J. Anim. Physiol. Anim. Nutr. (Berl). 96:825–833. doi:10.1111/j.1439-0396.2011.01231.x 21929727 10.1111/j.1439-0396.2011.01231.x

[CIT0065] Price S. B., Cheng C. M., Kaspar C. W., Wright J. C., DeGraves F. J., Penfound T. A., Castanie-Cornet M. P., and Foster J. W. 2000 Role of rpoS in acid resistance and fecal shedding of *Escherichia coli* O157:H7. Appl. Environ. Microbiol. 66:632–637. doi:10.1128/AEM.02980-15 10653728 10.1128/aem.66.2.632-637.2000 PMC91873

[CIT0066] Raya R. R., Oot R. A., Moore-Maley B., Wieland S., Callaway T. R., Kutter E. M., and Brabban A. D. 2011 Naturally resident and exogenously applied T4-like and T5-like bacteriophages can reduce *Escherichia coli* O157. Bacteriophage. 1:15–24. doi:10.4161/bact.1.1.14175 21687531 10.4161/bact.1.1.14175 PMC3109454

[CIT0067] Reyer H., Zentek J., Männer K., Youssef I. M. I., Aumiller T., Weghuber J., Wimmers K., and Mueller A. S. 2017 Possible molecular mechanisms by which an essential oil blend from star anise, rosemary, thyme, and oregano and saponins increase the performance and ileal protein digestibility of growing broilers. J. Agric. Food Chem. 65:6821–6830. doi:10.1021/acs.jafc.7b01925 28722406 10.1021/acs.jafc.7b01925

[CIT0068] Reyes-Jara A., Cordero N., Aguirre J., Troncoso M., and Figueroa G. 2016 Antibacterial effect of copper on microorganisms isolated from bovine mastitis. Front. Microbiol. 7:626. doi:10.3389/fmicb.2016.00626 27199953 10.3389/fmicb.2016.00626 PMC4848319

[CIT0069] Richard D., Ravigné V., Rieux A., Facon B., Boyer C., Boyer K., Grygiel P., Javegny S., Terville M., Canteros B. I., et al 2017 Adaptation of genetically monomorphic bacteria: evolution of copper resistance through multiple horizontal gene transfers of complex and versatile mobile genetic elements. Mol. Ecol. 26:2131–2149. doi:10.1111/mec.14007 28101896 10.1111/mec.14007

[CIT0070] Ricke S. C 2003 Perspectives on the use of organic acids and short chain fatty acids as antimicrobials. Poult. Sci. 82:632–639. doi:10.1093/ps/82.4.632 12710485 10.1093/ps/82.4.632

[CIT0071] Riley M. A., and Wertz J. E. 2002 Bacteriocins: evolution, ecology, and application. Annu. Rev. Microbiol. 56:117–137. doi:10.1146/annurev.micro.56.012302.161024 12142491 10.1146/annurev.micro.56.012302.161024

[CIT0072] Rivas L., Coffey B., McAuliffe O., McDonnell M. J., Burgess C. M., Coffey A., Ross R. P., and Duffy G. 2010 In vivo and ex vivo evaluations of bacteriophages e11/2 and e4/1c for use in the control of *Escherichia coli* O157:H7. Appl. Environ. Microbiol. 76:7210–7216. doi:10.1128/AEM.01530-10 20851992 10.1128/AEM.01530-10 PMC2976219

[CIT0073] Rosander A., Connolly E., and Roos S. 2008 Removal of antibiotic resistance gene-carrying plasmids from *Lactobacillus reuteri* ATCC 55730 and characterization of the resulting daughter strain, *L. reuteri* DSM 17938. Appl. Environ. Microbiol. 74:6032–6040. doi:10.1128/AEM.00991-08 18689509 10.1128/AEM.00991-08 PMC2565949

[CIT0074] Rosen B. P 2002 Transport and detoxification systems for transition metals, heavy metals and metalloids in eukaryotic and prokaryotic microbes. Comp. Biochem. Physiol. A. Mol. Integr. Physiol. 133:689–693. doi:10.1016/S1095-6433(02)00201-5 12443926 10.1016/s1095-6433(02)00201-5

[CIT0075] Rozema E. A., Stephens T. P., Bach S. J., Okine E. K., Johnson R. P., Stanford K., and McAllister T. A. 2009 Oral and rectal administration of bacteriophages for control of *Escherichia coli* O157:H7 in feedlot cattle. J. Food Prot. 72:241–250. doi:10.4315/0362-028X-72.2.241 19350968 10.4315/0362-028x-72.2.241

[CIT0076] Rudramurthy G. R., Swamy M. K., Sinniah U. R., and Ghasemzadeh A. 2016 Nanoparticles: alternatives against drug-resistant pathogenic microbes. Molecules. 21:1–31. doi:10.3390/molecules21070836 10.3390/molecules21070836 PMC6273897 27355939

[CIT0077] Ruparelia J. P., Chatterjee A. K., Duttagupta S. P., and Mukherji S. 2008 Strain specificity in antimicrobial activity of silver and copper nanoparticles. Acta Biomater. 4:707–716. doi:10.1016/j.actbio.2007.11.006 18248860 10.1016/j.actbio.2007.11.006

[CIT0078] Ryan M. P., Meaney W. J., Ross R. P., and Hill C. 1998 Evaluation of lacticin 3147 and a teat seal containing this bacteriocin for inhibition of mastitis pathogens. Appl. Environ. Microbiol. 64:2287–2290. PMCID: PMC106317. 9603853 10.1128/aem.64.6.2287-2290.1998 PMC106317

[CIT0079] Saez A. C., Zhang J., Rostagno M. H., and Ebner P. D. 2011 Direct feeding of microencapsulated bacteriophages to reduce *Salmonella* colonization in pigs. Foodborne Pathog. Dis. 8:1269–1274. 21854261 10.1089/fpd.2011.0905

[CIT0080] Salim H. M., Kang H. K., Akter N., Kim D. W., Kim J. H., Kim M. J., Na J. C., Jong H. B., Choi H. C., Suh O. S., et al 2013 Supplementation of direct-fed microbials as an alternative to antibiotic on growth performance, immune response, cecal microbial population, and ileal morphology of broiler chickens. Poult. Sci. 92:2084–90. doi:10.3382/ps.2012–02947 23873556 10.3382/ps.2012-02947

[CIT0081] Stanford K., McAllister T. A., Niu Y. D., Stephens T. P., Mazzocco A., Waddell T. E., and Johnson R. P. 2010 Oral delivery systems for encapsulated bacteriophages targeted at *Escherichia coli* O157:H7 in feedlot cattle. J. Food Prot. 73:1304–1312. doi: 10.4315/0362-028X-73.7.1304 20615343

[CIT0082] Tremblay Y. D., Caron V., Blondeau A., Messier S., and Jacques M. 2014 Biofilm formation by coagulase-negative staphylococci: impact on the efficacy of antimicrobials and disinfectants commonly used on dairy farms. Vet. Microbiol. 172:511–518. doi:10.1016/j.vetmic.2014.06.007 24984943 10.1016/j.vetmic.2014.06.007

[CIT0083] Upadhaya S. D., Lee K. Y., and Kim I. H. 2014 Protected organic acid blends as an alternative to antibiotics in finishing pigs. Asian-Australas. J. Anim. Sci. 27:1600–1607. doi:10.5713/ajas.2014.14356 25358320 10.5713/ajas.2014.14356 PMC4213705

[CIT0084] Uyeno Y., Shigemori S., and Shimosato T. 2015 Effect of probiotics/prebiotics on cattle health and productivity. Microbes Environ. 30:126–132. doi:10.1264/jsme2.ME14176 26004794 10.1264/jsme2.ME14176 PMC4462921

[CIT0085] Vahjen W., Pietruszyńska D., Starke I. C., and Zentek J. 2015 High dietary zinc supplementation increases the occurrence of tetracycline and sulfonamide resistance genes in the intestine of weaned pigs. Gut Pathog. 7:23. doi:10.1186/s13099-015-0071-3 26322131 10.1186/s13099-015-0071-3 PMC4551370

[CIT0101] Van Boeckel T. P., Brower C., Gilbert M., Grenfell B. T., Levin S. A., Robinson T. P., Teillant A., and Laxminarayan R. 2015 Global trends in antimicrobial use in food animals. Proc. Natl. Acad. Sci. 112:5649–5654. doi:10.1073/pnas.1503141112 25792457 10.1073/pnas.1503141112 PMC4426470

[CIT0102] Ventola C. L. 2015 The antibiotic resistance crisis: part 1: causes and threats. P T. A peer-reviewed. J. Formul. Manag. 40:277–283. PMCID: PMC4378521; PMID: 25859123. https://www.ncbi.nlm.nih.gov/pmc/articles/PMC4378521/pdf/ptj4004277.pdf PMC4378521 25859123

[CIT0086] Wagner P. L., and Waldor M. K. 2002 Bacteriophage control of bacterial virulence. Infect. Immun. 70:3985–3993. doi:10.1128/IAI.70.8.3985 12117903 10.1128/IAI.70.8.3985-3993.2002 PMC128183

[CIT0087] Wang X., Liu S., Li M., Yu P., Chu X., Li L., Tan G., Wang Y., Chen X., Zhang Y., and Ning C. 2016 The synergistic antibacterial activity and mechanism of multicomponent metal ions-containing aqueous solutions against *Staphylococcus aureus*. J. Inorg. Biochem. 163:214–220. doi:10.1016/j.jinorgbio.2016.07.019 27569414 10.1016/j.jinorgbio.2016.07.019

[CIT0088] Woods E. J., Cochrane C. A., and Percival S. L. 2009 Prevalence of silver resistance genes in bacteria isolated from human and horse wounds. Vet. Microbiol. 138:325–329. doi:10.1016/j.vetmic.2009.03.023 19362435 10.1016/j.vetmic.2009.03.023

[CIT0089] Xiu Z., Liu Y., Mathieu J., Wang J., Zhu D., and Alvarez P. J. 2014 Elucidating the genetic basis for *Escherichia coli* defense against silver toxicity using mutant arrays. Environ. Toxicol. Chem. 33:993–997. doi:10.1002/etc.2514 24408659 10.1002/etc.2514

[CIT0090] Yang C., Chowdhury M. A., Huo Y., and Gong J. 2015 Phytogenic compounds as alternatives to in-feed antibiotics: potentials and challenges in application. Pathogens. 4:137–156. doi:10.3390/pathogens4010137 25806623 10.3390/pathogens4010137 PMC4384076

[CIT0091] Yin Y., Gu J., Wang X., Song W., Zhang K., Sun W., Zhang X., Zhang Y., and Li H. 2017 Effects of copper addition on copper resistance, antibiotic resistance genes, and intl1 during swine manure composting. Front. Microbiol. 8:1–10. doi:10.3389/fmicb.2017.00344 28316595 10.3389/fmicb.2017.00344 PMC5335643

[CIT0096] Zaman S. B., Hussain M. A., Nye R., Mehta V., Mamun K. T., and Hossain N. 2017 A review on antibiotic resistance: alarm bells are ringing. Cureus. 9. doi:10.7759/cureus.1403. http://www.cureus.com/articles/7900-a-review-on-antibiotic-resistance-alarm-bells-are-ringing 10.7759/cureus.1403 PMC5573035 28852600

[CIT0092] Zhang D., Li R., and Li J. 2012 *Lactobacillus reuteri* ATCC 55730 and L22 display probiotic potential in vitro and protect against Salmonella-induced pullorum disease in a chick model of infection. Res. Vet. Sci. 93:366–373. doi:10.1016/j.rvsc.2011.06.020 21764090 10.1016/j.rvsc.2011.06.020

[CIT0093] Zhou H., Fang J., Tian Y., and Lu X. Y. 2014 Mechanisms of nisin resistance in Gram-positive bacteria. Ann. Microbiol. 64:413–420. doi:10.1007/s13213-013-0679-9

